# Effect of Polydextrose/Fructooligosaccharide Mixture on Constipation Symptoms in Children Aged 4 to 8 Years

**DOI:** 10.3390/nu13051634

**Published:** 2021-05-13

**Authors:** Mauro Sérgio Toporovski, Mauro Batista de Morais, Abrão Abuhab, Mauro Acir Crippa Júnior

**Affiliations:** 1 Faculdade de Ciências Médicas da Santa Casa de São Paulo, Responsável pela Disciplina de Gastroenterologia Pediátrica, São Paulo 01234-001, Brazil; 2 Disciplina de Gastroenterologia Pediátrica, Escola Paulista de Medicina (EPM), Universidade Federal de São Paulo (UNIFESP), São Paulo 04023-062, Brazil; maurobmorais@gmail.com; 3 Hypera Pharma, São Paulo 05676-120, Brazil; abrao.abuhab@hypera.com.br; 4 Allergisa-Contract Research Organization, Campinas 13084-791, Brazil; mauro.crippa@grupoinvestiga.com.br

**Keywords:** constipation, dietary fiber, clinical trial

## Abstract

Constipation is a frequent problem in children. We evaluated the effect of a mixture (polydextrose [PDX] and fructooligosaccharide [FOS]) in children with constipation. We performed a prospective interventional study with a mixture (PDX 4.17 g and FOS 0.45 g) in a daily dose of food supplement. The intervention lasted 45 days, with visits at 15, 30, and 45 days after administration. The sample comprised 105 patients, of whom 77 completed the intervention. A statistically significant reduction in the frequency of symptoms was observed at the end of the study. The frequency of children with fewer than three bowel movements per week dropped from 59.7% to 11.7%, and there was a decrease in the frequency of Bristol type 1 and 2 dry stools (68.8% to 7.8%), pain on defecation (79.2% to 10.4%), and fear of defecation (68.8% to 3.9%). The proportion of children with abdominal pain symptoms decreased from 84.2% to 2.6% at the end of the study. A relevant limitation of the present study was the lack of a control group treated with placebo. The administration of the PDX/FOS mixture was accompanied by a significant reduction in the frequency of constipation symptoms of the children evaluated. The tolerability was very good, and the rate of adverse effects was low.

## 1. Introduction

Constipation is a frequent problem among children in Brazil and other countries, accounting for as much as a quarter of elective gastroenterological visits [1,2]. The prevalence of constipation ranges from 14.7% to 38.8% in the pediatric population [3]. This wide range results from the diagnostic criteria used, the geographic region studied, and the age of the participants [3,4]. Studies have shown a higher prevalence of constipation in children of preschool age, with no difference between the sexes [4,5].

For children older than 4 years of age, functional constipation is currently defined by the Rome IV criteria (2016) as the occurrence of at least two of the following clinical manifestations for longer than 1 month: two or fewer bowel movements per week, at least one episode of fecal incontinence per week, pain on defecation, retentive posturing, defecation of stools that can obstruct the toilet, and the presence of fecal mass in the rectum [6]. In more than half of cases, functional constipation begins before 2 years of age, but there is a frequent delay in its diagnosis and in the implementation of dietary and drug interventions, leading to greater severity and a worsening of the prognosis [1,7,8].

According to some of the best-known pediatric guidelines, the treatment of constipation involves guidance on bowel retraining, correction of dietary problems, increased intake of dietary fiber and fluids, disimpaction of fecalomas, and maintenance drug therapy. In recent years, special emphasis has been given to the use of osmotic products because they provide a more adequate hydration of the fecal bolus, increase intestinal transit, and are generally well tolerated. Polyethylene glycol (PEG) 3350 with or without electrolytes and PEG 4000 are the preferred drugs, and lactulose is the second therapeutic option. Mineral oil and magnesium hydroxide are considered second-line therapies, although they are restricted in certain age groups and can induce serious adverse effects, such as mineral oil aspiration [8,9]. These guidelines state that dietary fibers are not useful for treating functional constipation. However, they do not make specific recommendations for prevention, mild cases, and children who have isolated clinical manifestations of functional constipation but do not fulfill the Rome criteria. The guidelines of the World Gastroenterology Organisation, on the other hand, recommend a cascade of options starting with dietary advice (fiber and fluid) and fiber supplementation as the first step for non-complicated chronic constipation in adults [10].

PDX/FOS can provide osmotic effects in the intestinal lumen. These effects may help prevent and treat mild clinical manifestations of constipation. Galactooligosaccharides (GOS), fructooligosaccharides (FOS), and polydextrose (PDX) have positive effects in the treatment of constipation, with virtually no adverse effects. Even so, few pediatric clinical trials have evaluated products with these fibers [11,12,13,14]. Similarly, there have been no clinical trials evaluating the effect on stool consistency and shape as defined in the Bristol stool scale [15]. 

Therefore, we hypothesized that a PDX/FOS mixture might be useful as an initial treatment in children with mild to moderate functional constipation or with isolated clinical manifestations of functional constipation who do not fulfill the Rome criteria. The objective of this study was to evaluate the effect of a PDX/FOS mixture on the clinical manifestations of constipation in children aged 4 to 8 years. The adherence to this mixture and its tolerability and safety were also evaluated.

## 2. Materials and Methods

### 2.1. Study Design

This was a prospective, noncomparative, interventional study on the effect of a PDX/FOS mixture on the control of clinical manifestations of constipation in children. After recruitment, patients were monitored for 2 weeks to confirm the presence of gastrointestinal clinical manifestations suggestive of constipation (pre-intervention period). The intervention lasted 45 days. The effect was analyzed in three periods of intervention: 1. From 0 to 15 days, 2. From 16 to 30 days, and 3. From 31 to 45 days. Stool samples were collected before the beginning of the intervention and at the end of the observation period.

### 2.2. Sample

One hundred and twenty eight children between 4 and 8 years of age comprised the initial study population and had symptoms related to constipation for at least 1 month prior to the first visit, which characterized the chronicity of the reported constipation symptoms. 

After that, there was an additional 15-day period of follow-up. During this period, the inclusion criteria were confirmed. These criteria were the report of one or more of the following clinical manifestations: pain on defecation, fear of defecation, decreased weekly frequency of bowel movements (fewer than three per week), hard stools, pebble-shaped or cylindrical and thick stools with cracks (types 1 and 2 on the Bristol scale), and abdominal pain. Twenty-three children did not meet the above criteria and did not continue the clinical trial. The sample comprised children between the ages of 4 and 8 years who had sphincter control. These criteria were defined according to previous suggestions for diagnosing functional constipation [10,15,16]. Participants of both sexes were included. An informed consent form was signed by each child’s guardian, and an assent form was signed by literate children. The study was conducted according to the guidelines of the Declaration of Helsinki, and approved by the Investiga Ethics Committee/Institutional Review Board CAAE: 85449618.5.0000.5599, approved on 27 March 2018.

The following exclusion criteria were considered: use of antibiotics or medications with laxative effects in the previous 30 days, other digestive diseases, uncontrolled nondigestive comorbidities such as allergic diseases, and history of any type of abdominal surgery.

### 2.3. Intervention

The intervention consisted of the daily administration of 10 mL of a food supplement containing a mixture of 4.17 g of PDX and 0.45 g of FOS. The amount of the product was measured in a measuring cup. The content of each bottle was 240 mL. The final composition was as follows: water, 53.53%; polydextrose, 41.7%; and FOS, 4.47%. Citric acid, sodium citrate, potassium sorbate, sodium benzoate, and strawberry flavor accounted for less than 1%. After recruitment, the parents received a form to be filled out daily.

At the evaluation visits (days 15, 30, and 45), information on bowel habits and clinical manifestations of constipation (number of bowel movements, presence or absence of pain on defecation, presence or absence of abdominal pain, fear of evacuation, and stool type classified according to the Bristol scale) was recorded as reported by parents. The tolerability and volume of the product consumed were measured, and the occurrence of adverse effects was recorded.

Stool characteristics were evaluated using the seven-category Bristol scale. The parents and guardians were instructed to photograph the children’s feces at least twice a week and send the images by telephone to the research center. At the research center, the photos were independently classified into the seven categories of the Bristol scale. Subsequently, the episode with the driest stool recorded in the observation period was assigned to each period for each patient. Bristol types 1 and 2 stools were considered to be dry, and stool types were divided into two groups (type 1 or 2 and type 3, 4, 5, 6, or 7) for statistical analysis.

Stool samples were collected from 30 patients before and in the last week of the intervention with the mixture. Families brought the bottles to measure the product consumption at each medical evaluation. Fecal samples were stored refrigerated (4 °C). On delivery, they were homogenized to observe the color and appearance. Aliquots were taken for the measurement of fecal pH using a paper-strip colorimetric method.

For the statistical analysis, demographic data were expressed as mean and standard deviation (*t* test) and numbers and percentage (chi-square test). The clinical manifestations of constipation during the intervention were compared using the McNemar test and expressed as numbers and percentage. Bowel movements were compared using ANOVA followed by a Fisher’s least significant difference test. The frequency of adverse events in children who completed and did not complete the intervention was compared using the chi-square test and a Fisher’s exact test (nonparametric variables). The software applications XLSTAT 2019 (Addinsoft Inc. New York, NY, USA) and Minitab version 14 (Minitab, LLC. State College, PA, USA) were used to perform the calculations. *p* values lower than 0.05 were considered statistically significant.

## 3. Results

A total of 128 children were recruited. The presence of clinical manifestations of constipation in 23 (18.0%) children during the 2 weeks before the start of the intervention was not confirmed. Of the 105 children who started the intervention, 28 (26.7%) did not complete the full intervention period for the following reasons: violation of the protocol, two patients; interruption of the intervention, 12 patients; and not attending the visits for clinical reassessment, 14 patients.

Table 1 shows that there were no statistically significant differences in age, sex, or clinical characteristics between children who completed the intervention (*n* = 77) and those who dropped out (*n* = 28), except for the higher frequency of type 1 and 2 stools in the group that completed the intervention.

**Figure 1:**
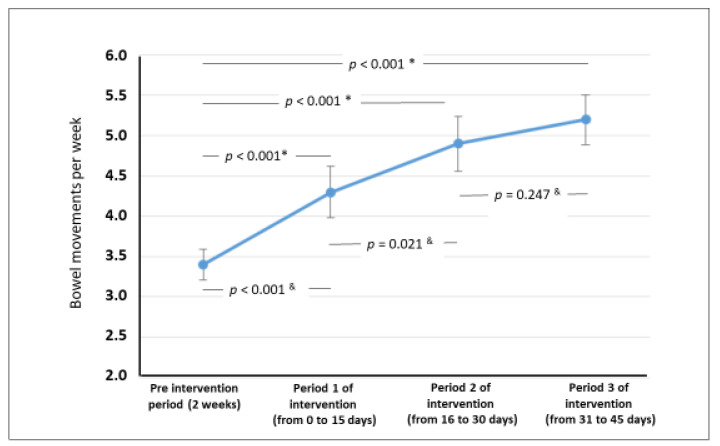
Bowel movements per week during the pre-intervention period and in the three periods * comparison versus pre-intervention, ^&^ for comparisions between each subsequent period ANOVA (Fisher’s least significant difference test).

Figure 1 shows that there was a statistically significant increase in the weekly frequency of bowel movements throughout the three intervention periods. There was a statistically significant increase in all evaluations performed at day 45 compared with the beginning of the intervention. PDX/FOS significantly reduced the proportion of children with fewer than three bowel movements per week, defecation of Bristol type 1 and 2 stools, pain on defecation, fear of defecation, and abdominal pain (Table 2).

**Figure 2:**
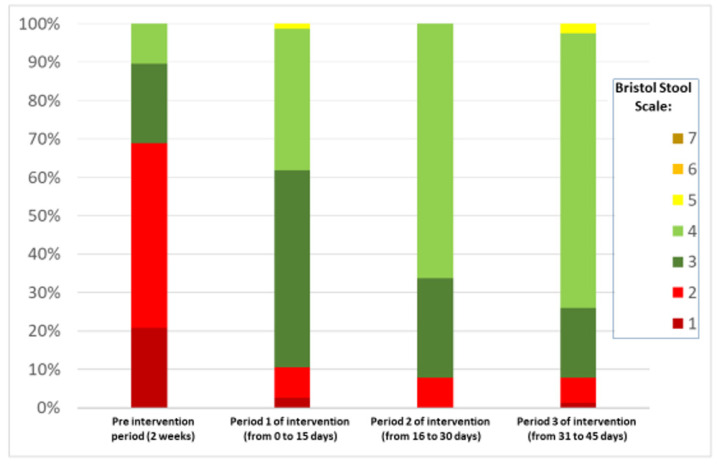
Bristol stool type percentages in the pre-intervention period and in periods 1, 2, and 3 of the intervention.

Figure 2 shows the change in the defecation pattern according to the Bristol scale. Before the intervention, there was a predominance of defecation of type 1 and 2 stools. As the intervention went on, there was a significant increase in mainly type 3 and 4 stools.

The adherence to the prescribed product was high: 99.4%, 99.6%, and 99.4% of the doses prescribed were taken in the three intervention periods (2 weeks each).

Table 3 shows the frequency of adverse events in the groups of children who completed and did not complete the intervention. Most adverse events occurred only once and were mild. The frequency of adverse events was similar for the patients who completed the intervention and those who did not at up to 2 weeks of intervention.

**Figure 3:**
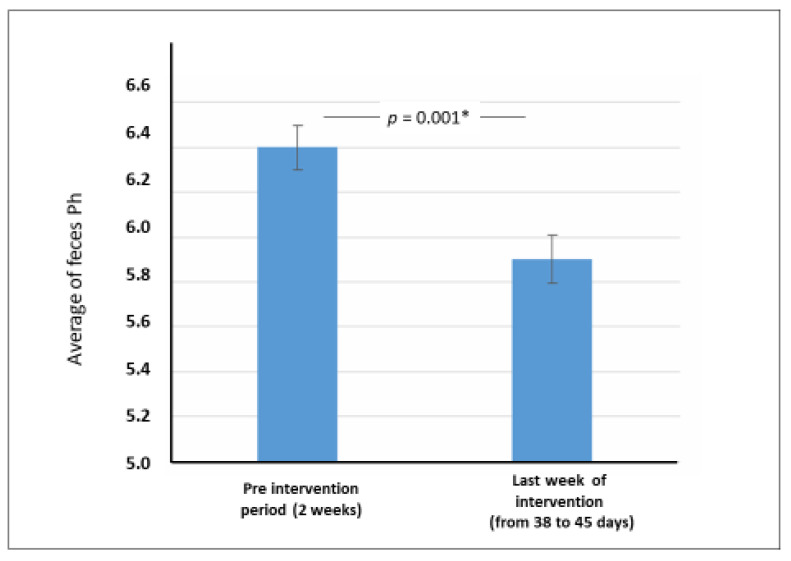
* Fecal pH in the pre-intervention period and in the last week of the intervention (mean and standard-deviation, paired *t* test)—*n* = 30.

Figure 3 shows adecrease in fecal pH between the pre-intervention period and the last week of the intervention (from 38 to 45 days).

## 4. Discussion

The results of the present study clearly show that the PDX/FOS mixture significantly decreased the frequency of clinical manifestations of constipation in children. The reduction in fecal pH suggests that at least a portion of the mixture may have been fermented in the intestinal lumen. The frequency of adverse events associated with the mixture was low and was not associated with the need to discontinue the supplement.

A lower dietary fiber intake is associated with a higher risk of constipation, although results on the efficacy of dietary fiber alone in treatment are controversial [1,2,3,4]. A systematic review and meta-analysis published in 2018 [17] evaluated nine randomized clinical trials on the use of dietary fiber in the treatment of constipation (total of 680 pediatric patients). The treatment with dietary fiber did not have a positive effect on the frequency of bowel movements, fecal incontinence, abdominal pain, or success of therapy. The results should be interpreted with caution owing to the heterogeneity and methodological limitations found in the clinical trials included.

The beneficial effect of mixtures on the composition of the intestinal microbiota has been highlighted in the literature, as evidenced in a review published in 2017 [18]. One noteworthy aspect is the growth of strains of the genera *Bifidobacterium* and *Lactobacillus*, which exert a fermentative effect by producing short-chain fatty acids, such as acetic acid, propionic acid, and butyric acid. This, in turn, leads to an increase in lactic acid production and a consequent decrease in fecal pH. In this context, a double-blind, placebo-controlled clinical trial of infants with constipation showed that the administration of FOS led to less uncomfortable defecation, the passing of softer stools, and a higher intestinal transit rate. This functional effect was characterized by an increased intestinal colonization by bacteria of the genus *Bifidobacteria* [12]. FOS is a dietary sugar and is incorporated into foods as a functional dietary fiber [14].

The present study was performed to analyze a PDX/FOS mixture. Polydextrose is a polysaccharide synthesized from dextrose that reaches the colon mostly intact, where it exerts osmotic effects. PDX is safe and does not usually cause adverse effects [11,19]. In our study, the PDX/FOS mixture led to a reduction in the percentage of children with fewer than three bowel movements per week (from 59.7% before the intervention to 11.7% at the end; *p* < 0.05). The weekly frequency of bowel movements increased from 3.4 to 5.2 (*p* < 0.001). Figure 1 shows an increase in the frequency of bowel movements as early as in the first return visit (day 15 of the PDX/FOS intervention).

Regarding the characteristics of the stools, as assessed using the Bristol scale, the present study showed that at the beginning of the study, 68.8% of the children had extremely hard and dry stools (types 1 and 2). In the first evaluation, 15 days after the beginning of the PDX/FOS intervention, only eight (10.5%) patients kept passing type 1 or 2 stools on the Bristol scale. This effect persisted until the end of the intervention period. A history of abdominal pain episodes was present in 84.4% of the sample at the beginning of the intervention. The frequency of abdominal pain decreased to 48.1% at day 15 of PDX/FOS use and to 2.6% at the end of the study.

Our results were similar to those of a clinical trial that used a double-blind, placebo-controlled design to evaluate the efficacy of a mixture (unspecified fiber, PDX, FOS, GOS) and combined with 10^9^ colony-forming units of *L. casei*, *L. rhamnosus*, *L. plantarum*, and *B. lactis* for children from Turkey with constipation [20]. The placebo group of the Turkish clinical trial showed no statistically significant decrease in any of the clinical manifestations of constipation [20]. 

A clinical trial conducted in Brazil [21] assessed the effects of a mixture of a yogurt with PDX (4.0 g) and *L. acidophilus* and *B. lactis* HN019 administered for 14 days to adults with chronic constipation. The mixture led to an increase in the frequency of bowel movements and a reduction in colonic transit time. Although our study was not controlled, the clinical response we observed seemed to be superior to that observed in the above-mentioned clinical trial, probably because of the greater doses of fibers used in our study relative to body weight (children versus adults).

Few pediatric studies analyze the effect of fibers in pediatric patients with constipation (see above). It is worth mentioning two further studies conducted in Brazil. Higher frequencies of bowel movements and softer stools were observed in a group of pediatric patients who received a mixture of fibers (fructooligosaccharides, inulin, gum arabic, starch, soy polysaccharide, and cellulose) than in patients who received a placebo. After discontinuing the use of laxative drugs, one-third of the patients needed to restart drug treatment; this rate was similar to those recorded in the intervention and control groups [22]. In another pediatric clinical trial, the efficacy of GOS in the treatment of constipation relative to placebo was confirmed [23].

In pediatric patients, pain on defecation and retentive posturing (fear of evacuation) are manifestations involved in the onset and chronicity of constipation. These manifestations are part of the vicious cycle of pain made up of the passage–retention of hard stools–pain on evacuation. The interruption of this vicious cycle is considered essential to the control of constipation. Thus, the resolution of pain on defecation and retentive posturing is essential both for the success of the initial treatment and for the prevention of the recurrence of clinical manifestations upon dose reduction and discontinuation of osmotic drugs [7,22,24]. In our study, pain on, and fear of, defecation decreased significantly during the intervention with the PDX/FOS mixture (Table 2). Retentive posturing, expressed as fear of defecation, decreased from 68.8% to 3.9% at the end of follow-up (*p* < 0.005). The same finding was recorded for pain on defecation, which decreased from 79.3% to 10.4% (*p* < 0.005). It is worth noting that, unlike our study, the clinical trial conducted in Turkey [20] with a lower dose of mixture did not lead to a statistically significant reduction in retentive posturing, which occurred in 40.3% of their patients at the beginning of the intervention and in 31.9% at the end of the intervention (*p* = 0.251). Similarly, in the control group, the reduction in the occurrence of retentive posturing was not significant (from 51.4% to 45.9%; *p* = 0.316).

A relevant limitation of the present study was the lack of a control group treated with a placebo. The reason was that when the protocol was conceived the principal investigator considered that the comparison between the beginning and the end of the trial was more appropriate than giving a placebo for a very unpleasant symptom in children. In this regard, the data in Table 2 should be taken into account. The decrease of manifestations was higher than the expected for a placebo in a clinical trial of functional gastrointestinal disorder (around 30 percent) [25]. The decrease (see Table 2) in the percentage of children with less than three bowel movements per week was 48.0%; with types 1 and 2 of Bristol scale, 61.0%; fear of defecation, 64.9%; pain on defecation, 68.8%; and abdominal pain, 81.8%. Therefore, the present non-controlled clinical trial suggested the effectivity of a fiber mixture supplement in decreasing the clinical manifestation of functional constipation, that could be confirmed in future controlled trials. Besides, our data showed a decrease in fecal pH, suggesting that the clinical effect is related to the osmotic mechanism of action of the fiber mixture, which involves changes in the pattern of intestinal fermentation.

In conclusion, the mixture was well tolerated and adhered to by parents and accepted by children. It led to a significant decrease in the clinical manifestations of constipation associated with decreased fecal pH.

## Figures and Tables

**Table 1:** Comparison of the demographic and clinical characteristics of the trial participants.

	Completed the Intervention		
	Yes (*n* = 77)	No (*n* = 28)	*p*
Age	5.8 ± 1.3	5.9 ± 1.5	0.772 ^1^
Male sex	41 (53.2%)	19 (67.9%)	0.26 ^2^
Fewer than three bowel movements per week	46 (59.7%)	13 (46.4%)	0.32 ^2^
Bristol type 1 or 2 stools	53 (68.8%)	10 (35.7%)	0.004 ^2^
Abdominal pain	65 (82.9%)	19 (67.9%)	0.109 ^2^

^1^ Mean and standard deviation, *t* test. ^2^ Number and percentage, chi-square test.

**Table 2:** Clinical manifestations of constipation during the intervention with the mixture of fructooligosaccharides and polydextrose in 77 children.

	Pre-Intervention Period (2 Weeks)	Period 1 of Intervention (from 0 to 15 Days)		Period 2 of Intervention (from 15 to 30 Days)		Period 3 of Intervention (from 31 to 45 Days)	
	*N* (%)	*N* (%)	*p* *	*N* (%)	*p* *	*N* (%)	*p* *
Fewer than three bowel movements per week	46 59.7%	24 31.2%	<0.001	18 23.4%	<0.001	9 11.7%	<0.001
Type 1 or 2 stools on the Bristol scale	53 68.8%	8 10.5%	<0.001	6 7.8%	<0.001	6 7.8%	<0.001
Fear of defecation	53 68.8%	10 13.0%	<0.001	3 3.9%	<0.001	3 3.9%	<0.001
Pain on defecation	61 79.2%	18 23.4%	<0.001	8 10.4%	<0.001	8 10.4%	<0.001
Abdominal pain	65 84.4%	37 48.1%	<0.001	8 10.4%	<0.001	2 2.6%	<0.001

* McNemar test: Period 1, 2, or 3 of the intervention vs. pre-intervention period.

**Table 3:** Frequency of adverse events in children who completed and did not complete the intervention with the mixture of fructooligosaccharide and polydextrose.

	Completed the Intervention				
	Yes (*N* = 77)		No (*N* = 28)		*p*
	*N*	%	*N*	%	
Intensity					
Mild	17	22.1	8	28.6	0.665 ^1^
Moderate	1	1.3	3	10.7	0.571 ^2^
Frequency					
Once	11	14.3	9	32.1	0.075 ^1^
Recurrent	7	9.1	2	7.1	1.000 ^2^
Predicted adverse event					
Yes	8	10.4	3	10.7	1.000 ^2^
No	10	13.0	8	28.6	0.079 ^2^
Gastrointestinal					
Abdominal pain	4	5.2	4	14.3	0.204 ^2^
Diarrhea	3	3.9	0	0.0	0.562 ^2^
Flatulence/distension	1	1.3	1	3.6	0.464 ^2^
Nausea/vomiting	1	1.3	1	3.6	0.464 ^2^
Bleeding on defecation	2	2.6	0	0.0	1.000 ^2^
Nongastrointestinal					
Fever	2	2.6	0	0.0	1.000 ^2^
Upper airway symptoms	5	6.5	5	17.9	0.126 ^2^
Causal relationship					
Not related	5	6.5	4	14.3	0.242 ^2^
Unlikely	6	7.8	3	10.7	0.697 ^2^
Possible	7	9.1	3	10.7	0.724 ^2^
Probable	0	0.0	1	3.6	0.266 ^2^

^1^ Chi-square test. ^2^ Fisher’s exact test.
